# On myocardial siderosis and left ventricular dysfunction in hemochromatosis

**DOI:** 10.1186/1532-429X-15-24

**Published:** 2013-03-19

**Authors:** John-Paul Carpenter, Agata E Grasso, John B Porter, Farrukh Shah, James Dooley, Dudley J Pennell

**Affiliations:** 1 Royal Brompton and Harefield NHS Foundation Trust, Sydney Street, London, SW3 6NP, UK; 2 Imperial College, London, UK; 3 University College Hospital London, London, UK; 4 Whittington Hospital, London, UK; 5 University College London Medical School (Royal Free Campus), London, UK

**Keywords:** Iron overload, Heart, Hemochromatosis, Cardiomyopathy, Heart failure, Magnetic resonance

## Abstract

**Background:**

Chronically increased intestinal iron uptake in genetic hemochromatosis (HC) may cause organ failure. Whilst iron loading from blood transfusions may cause dilated cardiomyopathy in conditions such as thalassemia, the in-vivo prevalence of myocardial siderosis in HC is unclear, and its relation to left ventricular (LV) dysfunction is controversial. Most previous data on myocardial siderosis in HC has come from post-mortem studies.

**Methods:**

Cardiovascular magnetic resonance (CMR) was performed at first presentation of 41 HC patients (58.9 ±14.1 years) to measure myocardial iron and left ventricular (LV) ejection fraction (EF).

**Results:**

In 31 patients (*genetically confirmed* HFE-HC), the HFE genotype was C282Y/C282Y (n = 30) and C282Y/H63D (n = 1). Patients with other genotypes (n = 10) were labeled *genetically unconfirmed* HC. Of the *genetically confirmed* HFE-HC patients, 6 (19%) had myocardial siderosis (T2* <20 ms). Of these, 5 (83%) had heart failure and reduced LVEF which was correlated to the severity of siderosis (R^2^ 0.57, p = 0.049). Two patients had follow-up scans and both had marked improvements in T2* and LVEF following venesection. Myocardial siderosis was present in 6/18 (33%) of patients with presenting ferritin ≥1000 μg/L at diagnosis but in 0/13 (0%) patients with ferritin <1000 μg/L (p = 0.028). Overall however, the relation between myocardial siderosis and ferritin was weak (R^2^ 0.20, p = 0.011). In the 10 *genetically unconfirmed* HC patients, 1 patient had mild myocardial siderosis but normal EF. Of all 31 patients, 4 had low LVEF from other identifiable causes without myocardial siderosis.

**Conclusion:**

Myocardial siderosis was present in 33% of newly presenting *genetically confirmed* HFE-HC patients with ferritin >1000 μg/L, and was the commonest cause of reduced LVEF. Heart failure due to myocardial siderosis was only found in these HFE-HC patients, and was reversible with venesection. Myocardial iron was normal in patients with other causes of LV dysfunction.

## Background

Genetic hemochromatosis (HC) is a common disorder in the Caucasian population, with a prevalence of 2–5 per thousand [1]. In adults, the C282Y HFE protein variant (tyrosine substitution for cysteine at amino acid 282) is the most important variant associated with HC, and most adult HC patients are C282Y homozygotes. The frequency of C282Y heterozygotes in the North American and European population is 0.9% and 9.2% respectively. In addition, there is the H63D HFE protein variant (aspartate substitution for histidine at amino acid 63) which is associated with HC in C282Y/H63D compound heterozygotes [2], and this mutation has a heterozygote frequency in North America of 23% and 22% in Europe. An S65C mutation (cysteine substitution for serine at amino acid 65) has recently been suggested to be associated with a milder form of HC and carrier frequencies in Caucasian population are 1-4% [3,4]. Biochemical penetrance, with raised iron parameters such as ferritin and transferrin saturation, is 38-76% [5], but clinical penetrance is lower at 2-38% in men and 1-10% in women [6,7]. The risk of developing overt disease is modulated by other factors including alcohol, inflammation and viral infections [8,9]. Abnormal iron handling with HFE gene and other rarer genetic mutations causing HC, appears to be mediated via inappropriately low production of hepcidin, which is a master iron regulator [10]. This results in augmented iron export by ferroportin from duodenal enterocytes and reticuloendothelial macrophages, which chronically increases iron absorption from the duodenum and upper intestine, raises plasma iron levels and causes iron accumulation in the body [11].

A reported complication of excess iron deposition in HC is the development of dilated cardiomyopathy (DCM) [12]. Cardiovascular magnetic resonance (CMR) has been used to assess myocardial iron deposition and DCM in patients with thalassemia using the relaxation parameters T2* and T2, but T2* is in wider clinical use because of ease of acquisition and implementation [13,14]. Heart T2* falls with increasing iron loading [15,16], with the normal myocardial T2* being approximately 40 ms. Myocardial iron overload is present when T2* <20 ms [13], and heart failure usually only occurs when myocardial T2* is <10 ms [17]. However, while myocardial siderosis and DCM have been directly linked in thalassemia, and reversal of left ventricular (LV) dysfunction demonstrated with iron chelation [18], there is scant data on in-vivo myocardial iron loading in HC, and most knowledge is based on highly selected autopsy studies [19], with considerable controversy over the causation of LV dysfunction. Therefore, in this study we investigated the hypothesis that LV dysfunction was related to myocardial siderosis in HC.

## Methods

We prospectively studied 41 patients by CMR at the time of their first presentation with a clinical diagnosis of HC. In all patients, the clinical diagnosis was made in specialist HC clinics, and was based on accepted criteria, including characteristic clinical findings, abnormal serum parameters of iron metabolism (serum ferritin, serum iron, transferrin iron saturation) and HFE mutation analysis [20]. Genetic testing for the C282Y and H63D HFE mutations was performed in all patients. Patients had serum ferritin values taken at initial clinical presentation (and before commencing treatment with venesection in confirmed cases of HC) and this value was used as the presentation ferritin for comparisons with myocardial T2* and LV function.

CMR was performed on a 1.5T scanner (Siemens Sonata, Erlangen, Germany) with measurement of T2* of the heart and liver, and LV function, volumes and mass. In brief, the myocardial T2* images were acquired using a single 10mm-thick short-axis mid-ventricular slice of the LV with 8 echo times (2.6 to 16.7 ms, with 2.02 ms increment) in a single breath-hold [21]. A similar technique was used for the liver T2* acquisition, but with shorter echo times (0.9 ms to 16.0 ms, with 1.34 ms increment). Ventricular volumes, mass, and function were determined with the use of steady state free precession cines, with contiguous short-axis slices from base to apex [22]. For myocardial T2* measurement, a full thickness region of interest (ROI) was measured in the septum, distant from the cardiac veins and lungs to avoid susceptibility artefacts. A large ROI away from vessels was likewise analyzed for the liver. To derive T2*, the data was fitted with an exponential equation and a truncation model [23,24]. All myocardial T2* analyses were performed using Thalassemia-Tools, a plug-in of CMRtools (Cardiovascular Imaging Solutions, London, UK). Complete ventricular volumetric and mass analyses were also performed using CMRtools [22]. All data analysis was performed by a single experienced operator. The reproducibility of T2* and volume measurements have been reported previously [21,25].

Statistical analysis of normal variables is shown as mean ± standard deviation (SD) for data with a normal distribution, and median [Q1, Q3] for data with a non-normal distribution. Linear regression analysis was performed relating ejection fraction to myocardial T2*, and a p-value of <0.05 was considered statistically significant. Iron concentrations for liver and heart were calculated from the papers by Wood [26] and Carpenter [16]. This study was approved by the institutional Research Ethics Committee and all patients gave informed written consent. The study was carried out in accordance with the Declaration of Helsinki.

## Results

The homozygous C282Y/C282Y genotype was found in 30 patients with the compound heterozygous C282Y/H63D genotype found in 1 patient. These 31 patients were labeled as *genetically confirmed* HFE-HC [27]. The remaining 10 patients had other genotypes and were labeled as *genetically unconfirmed* HC (C282Y/wt in 5; H63D/H63D in 4; H63D/wt in 1). Further genetic testing for rare mutations in HFE and other iron handling genes was not performed. The baseline characteristics of the *genetically confirmed* HFE-HC and *genetically unconfirmed* HC patients are shown in Tables 1 and 2 respectively. The mean age of all 41 patients was 58.9 ±14.1 years indicating a middle-age to elderly population, and there was a strong male preponderance (73.1%). All patients had a raised ferritin level at presentation, and in addition the iron binding saturation or iron levels were also significantly raised. Median liver T2* was markedly reduced indicating liver iron loading with median liver iron concentration estimated at 8.7 mg/g dw [3.7, 18.3] in *genetically confirmed* HFE-HC patients, and 1.8 mg/g dw [1.3, 4.2] in *genetically unconfirmed* patients. Six patients underwent liver biopsy due to abnormal liver function test results, all of which showed fibrosis with cirrhotic change seen in three of the six. The median LV volumes, mass and EF were normal for the whole patient cohort, but were abnormal in the individual patients with impaired LV function.

**Table 1:** ***Genetically confirmed *HFE-HC patient group: patient characteristics at time of CMR scan**

Number of patients		31
Male/Female		25/6
Age (years): mean [SD]		58.8 [13.6]
Weight (kg): mean [SD]		80.8 [14.5]
Height (cm): mean [SD]		173 [11.8]
Race	Caucasian	31
Genetic mutation analysis: N (%)	C282Y/C282Y	30 (97%)
	C282Y/H63D	1 (3%)
Presentation ferritin (μg/L): median [Q1, Q3]; Normal 30-400		1286 [639, 2772]
Iron (μmol/L): mean [SD]; Normal 9-30		43.5 [12.7]
Iron binding saturation (%): mean [SD]; Normal 25-50		67.5 [19.8]
Hb (g/dL): mean [SD]; Normal 13-15		13.6 [1.4]
Liver T2* (ms): median [Q1, Q3]; Normal 26.7 ±4.2 ms		3.0 [1.4, 7.3]
Liver iron concentration (mg/g dw): median [Q1, Q3]		8.7 [3.7, 18.3]
LV EDV/BSA (mL/m^2^): median [Q1, Q3]; Normal 60-95		77 [64, 88]
LV ESV/BSA (mL/m^2^): median [Q1, Q3]; Normal 16-36		24 [20, 29]
LV EF (%): median [Q1, Q3]; Normal 58-76		70 [59, 73]
LV mass/BSA (g/m^2^): median [Q1, Q3]; Normal 53-84		70 [58, 90]
Cardiac T2* (ms): median [Q1, Q3]; Normal 40 ms		34.8 [25.4, 40.1]
Cardiac iron concentration (mg/g dw): median [Q1, Q3]		0.59 [0.50, 0.87]

(*BSA* body surface area, *EDV* end-diastolic volume, *EF* ejection fraction, *ESV* end-systolic volume, *Hb* Hemoglobin, *LV* left ventricle, *SD* standard deviation, *Q1* lower quartile, *Q3* upper quartile). Normal values from Westwood [21], and Maceira [22].

**Table 2:** ***Genetically unconfirmed *HC patient group: patient characteristics at time of CMR scan**

Number of patients		10	
Male/Female		6/4	
Age (years): mean [SD]		60.0 [15.9]	
Weight (kg): mean [SD]		70.4 [16.4]	
Height (cm): mean [SD]		175 [10.6]	
Race	Caucasian	10	
Genetic mutation analysis: N (%)	C282Y/wt	5 (50%)	
	H63D/H63D	4 (40%)	
	H63D/wt	1 (10%)	
Presentation ferritin (μg/L): median [Q1, Q3]; Normal 30-400		920 [660, 1912]	
Iron (μmol/L): mean [SD]; Normal 9-30		34.0 [11.6]	
Iron binding saturation (%): mean [SD]; Normal 25-50		64.8 [18.7]	
Hb (g/dL): mean [SD]; Normal 13-15		13.6 [1.4]	
Liver T2* (ms): median [Q1, Q3]; Normal 26.7 ±4.2 ms		16.4 [6.3, 23.0]	
Liver iron concentration (mg/g dw): median [Q1, Q3]		1.8 [1.3, 4.2]	
LV EDV/BSA (mL/m^2^): median [Q1, Q3]; Normal 60-95		79 [64, 83]	
LV ESV/BSA (mL/m^2^): median [Q1, Q3]; Normal 16-36		22 [19, 38]	
LV EF (%): median [Q1, Q3]; Normal 58-76		68 [63, 72]	
LV mass/BSA (g/m^2^): median [Q1, Q3]; Normal 53-84		80 [74, 89]	
Cardiac T2* (ms): median [Q1, Q3]; Normal 40 ms		30.9 [26.8, 34.5]	
Cardiac iron concentration (mg/g dw): median [Q1, Q3]		0.68 [0.60, 0.81]	

Abbreviations: wt - wild type; other abbreviations as Table 1.

**Figure 1:**
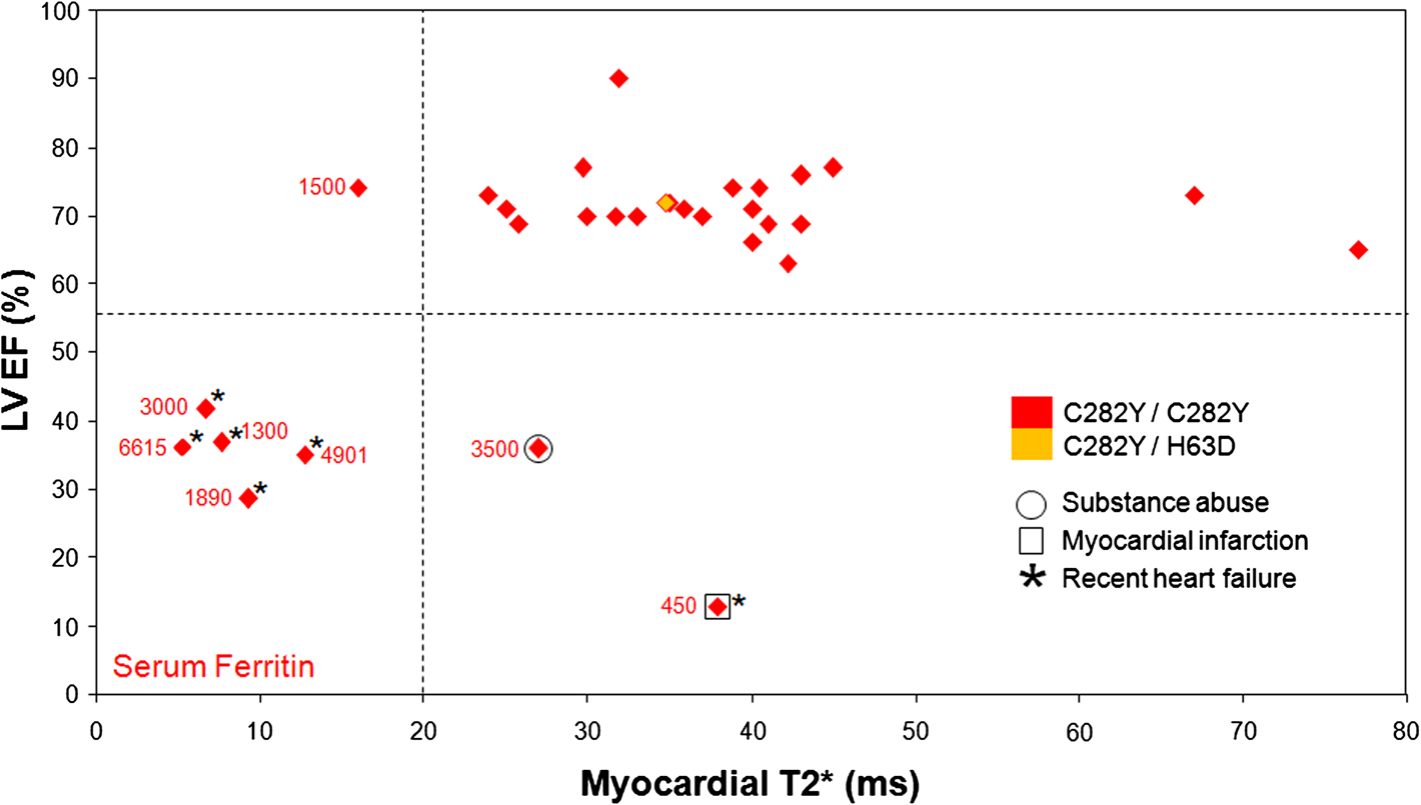
Relation between myocardial T2* and ejection fraction in *genetically confirmed *HFE-HC patients. The 2 patients with LV dysfunction and normal myocardial iron (T2* >20 ms) had clear alternative diagnoses for the impaired ejection fraction (as shown). The relation between myocardial T2* and LVEF is only significant when myocardial iron overload is present.

**Figure 2:**
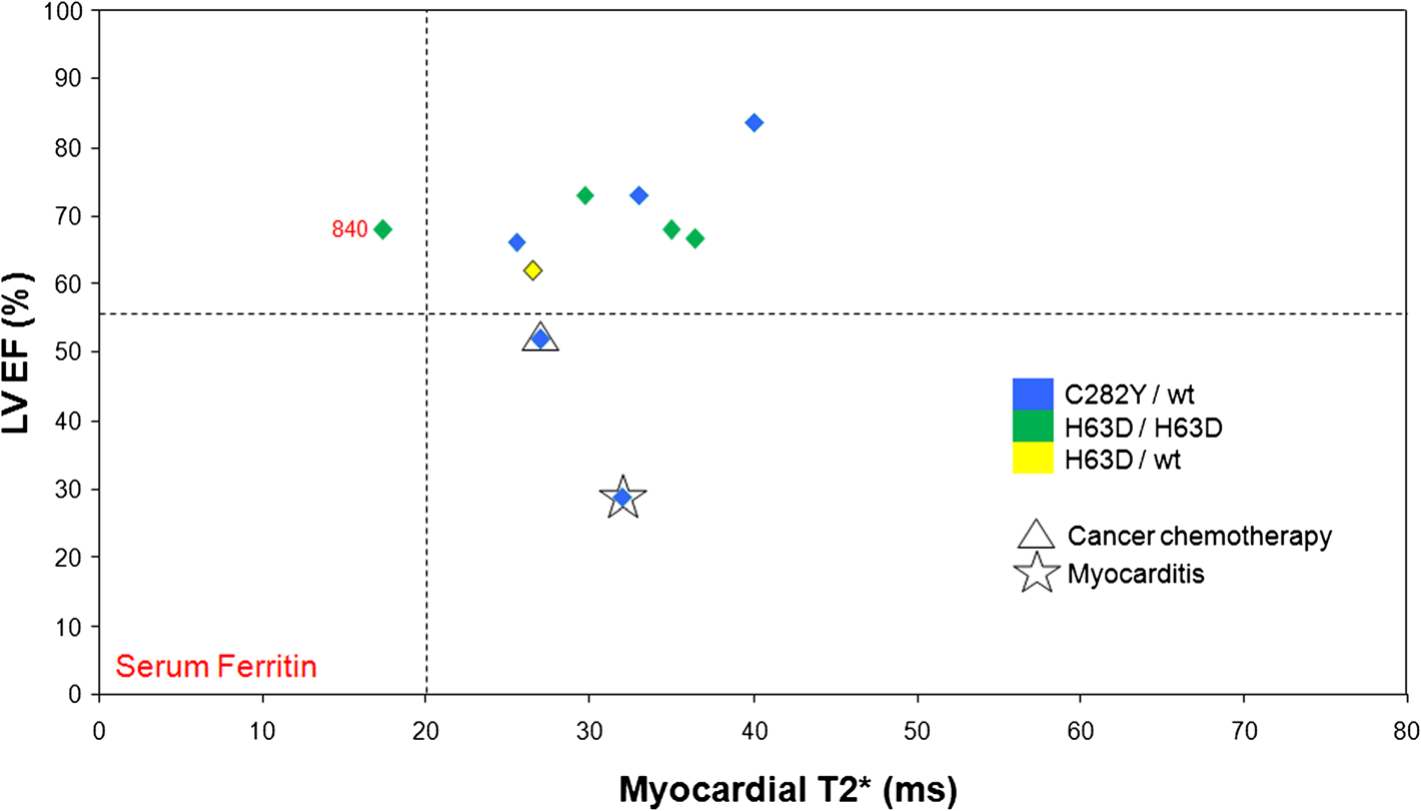
Relation between myocardial T2* and ejection fraction in *genetically unconfirmed *HC patients. The 2 patients with LV dysfunction and normal myocardial iron (T2* >20 ms) had clear alternative diagnoses for the impaired ejection fraction (as shown).

The results of the myocardial T2* scan at presentation of the patients in relation to the LV ejection fraction (EF) are shown in Figure 1 for the *genetically confirmed* HFE-HC patients, and Figure 2 for the *genetically unconfirmed* patients. Median time from clinical diagnosis to CMR T2* scan was 3.0 [0.25, 10.8] months, and from start of venesection to CMR scan was 0 [0, 3] months. Of the 31 *genetically confirmed* HFE-HC patients, 23 (74%) patients had both normal myocardial T2* and LV function. Six (19%) patients had a myocardial T2* below 20 ms indicating myocardial siderosis, and of these, 5 (83%) also had LV dysfunction, all of whom had a history of heart failure. All patients with myocardial siderosis had a serum ferritin at presentation >1300 ng/L (sensitivity 100%, specificity 64.0%). Two other patients had normal myocardial T2* (>20 ms) with reduced LVEF. Both these patients had a prior definitive diagnosis and documented clinical history which explained the LV dysfunction, namely chronic substance abuse with drugs and alcohol [1] and previous anterior myocardial infarction with severe three vessel disease at coronary angiography [1]. Of the 5 patients with recent heart failure who did not have an alternative explanation for their LV dysfunction, the investigations showed: serum ferritin 3000 [1890, 4901] μg/L, myocardial T2* 7.4 [6.7, 9.8] ms, liver T2* 1.2 [1.2,1.4] ms, LVEDV 236 ±70 ml, LVESV 161 ±56 ml, EF 35 ±5% and LV mass 161 ±56 g.

**Figure 3:**
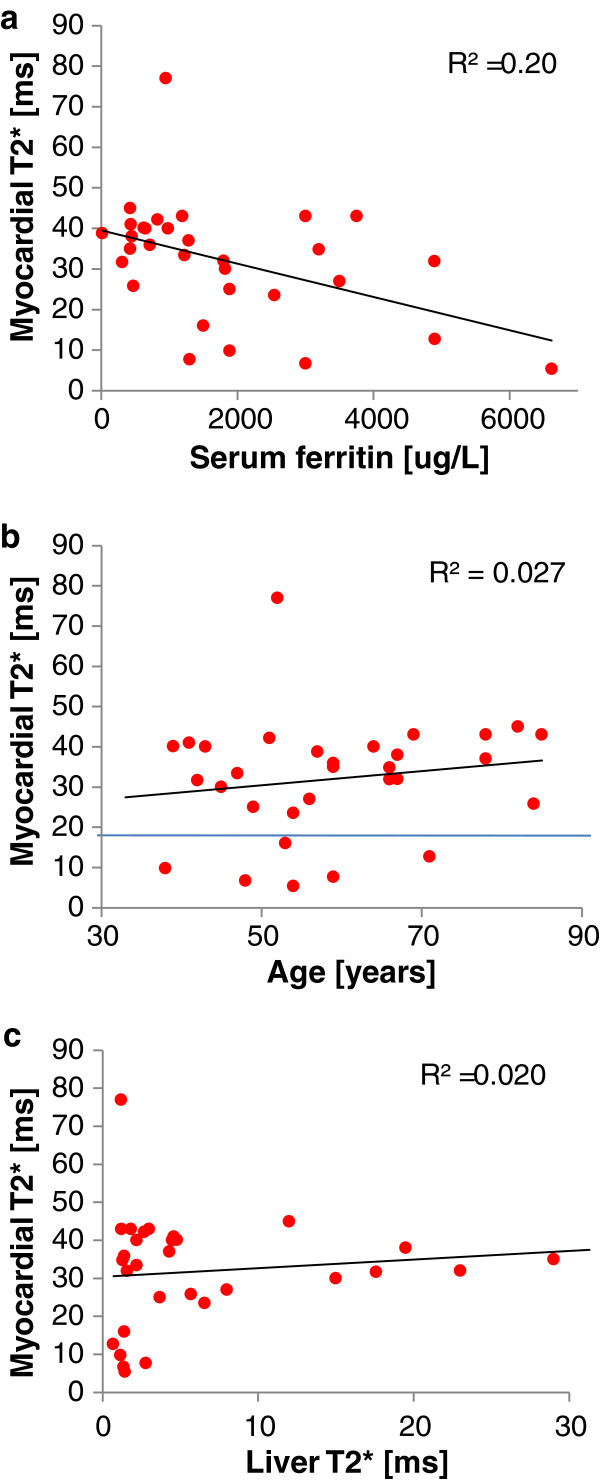
Relation between myocardial T2* and potential predictors of myocardial iron loading in the *genetically confirmed *HFE-HC patients: a) presentation serum ferritin (p = 0.011), b) age (p = 0.38), c) liver iron as measured by liver T2* (p = 0.44).

In order to examine whether there were any predictive measures of myocardial iron loading, regression analysis was performed of myocardial T2* against presentation serum ferritin, age and liver iron (as measured by liver T2*) as shown in Figure 3. There was a weak relation between increasing myocardial siderosis and increasing presentation serum ferritin (R^2^ 0.20, p = 0.011). Dividing serum ferritin by age yielded a slightly stronger correlation (R^2^ 0.29, p = 0.0017) against myocardial T2* than for ferritin alone. There were no statistically significant relations found for the regression of age (R^2^ 0.027, p = 0.38), and liver iron as measured by liver T2* (R^2^ 0.020, p = 0.44). Similarly, no correlation was found between myocardial siderosis and liver T2* divided by age (equivalent of the hepatic iron index: R^2^ 0.015, p = 0.51). Regression analysis showed no relation between myocardial T2* and EF for normal values of myocardial iron (T2* >20 ms; R^2^ 0.001; p = 0.85). However, for those patients with myocardial siderosis (T2* <20 ms), the relation between myocardial T2* and EF was significant (R^2^ 0.57; p = 0.049). All C282Y homozygotes with myocardial siderosis had liver T2* <3 ms (sensitivity 100%, specificity 64.0%).

Of the 10 *genetically unconfirmed* HC patients, 7 (70%) patients had both normal myocardial T2* and LV function. One patient had a myocardial T2* below 20 ms (indicating mild myocardial siderosis) with a serum ferritin of 840 μg/L at presentation but normal LV function. Two other patients had normal myocardial T2* (>20 ms) with reduced LV EF. Both these patients had a prior definitive diagnosis and documented clinical history which fully explained the LV dysfunction, namely a) non-Hodgkins lymphoma with 3 years of chemotherapy and bone marrow transplantation with whole body radiation and b) myocarditis with mid-wall LV fibrosis shown with late gadolinium enhancement CMR.

Late gadolinium enhancement (LGE) imaging was not performed routinely in the patient cohort. However 7 patients had LGE imaging for clinical indications. Of 6 patients with *genetically confirmed* HFE-HC*,* two patients had LGE, the first of which was the patient with the documented anteroseptal myocardial infarction. The other patient had heart failure and low T2* (Figure 1), with mid-wall fibrosis (myocardial T2* 9.8 ms with a severely dilated ventricle and LV EF of 29%) and no prior history of cardiac pathology. In the patients with *genetically unconfirmed* HC, only 1 had LGE (the patient with a documented prior history of myocarditis), with mid-wall fibrosis (Figure 2).

**Figure 4:**
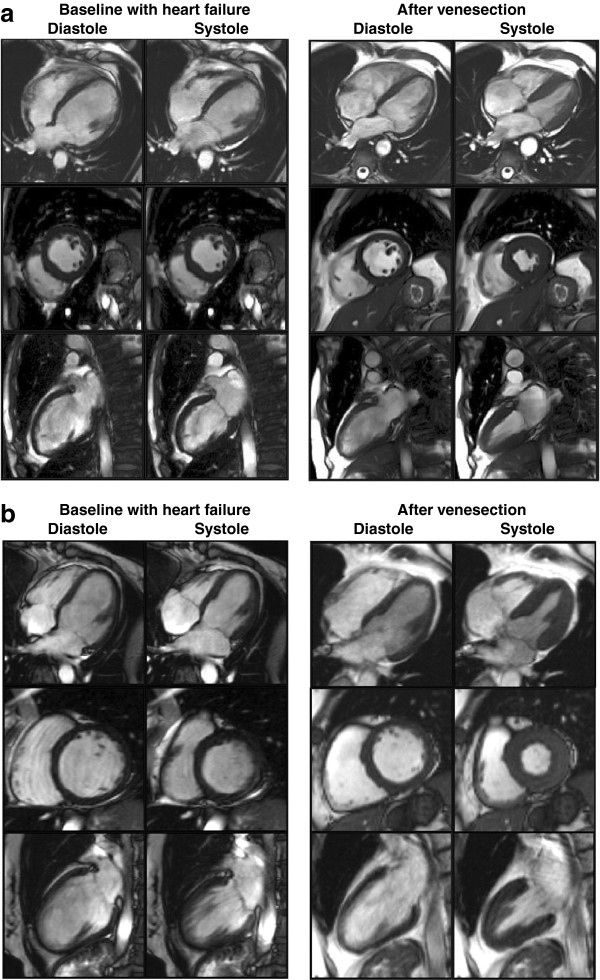
**Examples of CMR scans from newly presenting patients with C282Y homozygous hemochromatosis and heart failure. a**) 54 year-old male with presenting myocardial T2* of 5.4 ms and LVEF of 36%. When rescanned 31 months later after venesection, the cardiac T2* had improved to 15.7 ms, and the LVEF to 65%. **b**) 59 year-old female with presenting cardiac T2* of 7.4 ms and LVEF of 35%. Rescanning 19 months later after venesection showed improvement in T2* to 23.6 ms and LVEF to 61%. In both cases, venesection resulted in considerable improvement in iron loading and cardiac function. Top row: horizontal long axis, middle row: mid-ventricular short axis, bottom row: vertical long axis.

Two of the 5 patients with *genetically confirmed* HFE-HC who presented in heart failure were rescanned after venesection (Figure 4). Both repeat scans showed a considerable improvement in the LV EF and myocardial T2*.

## Discussion

In genetic hemochromatosis (HC), the intestinal absorption of iron is high and multi-organ iron overload with organ failure may occur over a period of decades. Myocardial iron loading is well known as a possible complication, but as far as we are aware, this is the first study of myocardial iron loading in newly presenting patients with possible HC. This is now feasible because of the non-invasive nature of T2* CMR as myocardial biopsy to assess iron loading is restricted to highly selected cases. Analysis of possible predictors of myocardial iron loading as assessed by T2* showed that all *genetically confirmed* HFE-HC patients with myocardial siderosis had a presenting serum ferritin value of >1000 μg/L, a value that is also associated with an increased risk of developing liver pathology. Above this threshold, myocardial siderosis was present in 6/18 (33%) patients compared with 0/13 (0%) patients with presenting ferritin values <1000 µg/L (p=0.028). Whilst the lowest serum ferritin measured in genetically confirmed HFE-HC patients with myocardial siderosis was 1300 ug/L, a more conservative threshold of 1000 ug/L would appear to be a more appropriate level for clinical practice, because the patient with the lowest serum ferritin of 1300 ug/L had a myocardial T2* of 7.4 ms indicating severe iron loading. The limited sample size of this study may therefore not have identified patients with a milder degree of myocardial iron loading associated with a lower serum ferritin. As this lower serum ferritin threshold would not miss patients with myocardial siderosis, it would seem reasonable on this basis of the data presented here to recommend T2* CMR on all confirmed HC patients presenting with a serum ferritin >1000 µg/L.

Another consideration in HC, is the development of heart failure. Dilated cardiomyopathy (DCM) is well recorded in HC, and reports recommend that serum ferritin should be measured in newly presenting cases of DCM [28]. In thalassemia, there is unequivocal evidence showing that iron causes the LV dysfunction, because severe myocardial siderosis is present in patients with heart failure [16,17], and LV dysfunction is reversible with iron chelation during which time the myocardial siderosis improves [18,29-31]. The relation between myocardial siderosis and LV dysfunction has never been shown in-vivo in HC. In addition the cause of LV dysfunction in HC is controversial. Apart from myocardial iron deposition, there have been 3 alternative proposed explanations.

The first alternative explanation for LV dysfunction in HC is that myocardial damage results from coronary artery disease (CAD) exacerbated by chronically increased iron levels. A correlation has been shown between high ferritin levels, LDL cholesterol and risk of myocardial infarction, with a plausible mechanism of augmented lipid peroxidation [32]. Iron could also promote atherogenesis and post-ischemic myocardial injury and perhaps arrhythmias [33]. Supportive data for this view includes the lower incidence of coronary artery disease in women due to lower iron levels through life resulting from menstruation, and the increased incidence of CAD in a cohort of 2,873 Framingham women who had a natural or surgical menopause. However, others have not found a relation between storage iron and myocardial infarction [34]. An additional issue in HC is the possible interaction of the genetic abnormality and risk of coronary disease. There has been a report of an increased risk of myocardial infarction in men who are carriers of the HFE C282Y mutation, which may suggest that that the mutation might confer an increased risk of CAD [35]. A study of 20,555 post menopausal women also showed that women with CAD risk factors who were heterozygous for the HFE C282Y mutation developed more CAD in comparison with matched women with homozygous wild-type HFE genotype [36]. These studies suggest that the HFE mutation in itself may promote CAD that could explain LV dysfunction.

The second alternative hypothesis is that the HFE gene is directly causative for DCM, with an increased frequency of the H63D mutation, but not the C282Y mutation [37]. The survival rate in this study was similar between the H63D carriers and non-carriers, but patients with the C282Y mutation had a shorter duration of illness before presentation, less LV volume dilatation and better fractional shortening. This is somewhat surprising because H63D has less of an effect on iron metabolism compared with C282Y, which suggests that factors other than just direct iron damage may predispose the patients to DCM. However, other studies have not observed differences in frequency between the DCM population with C282Y or H63D mutations and non carriers [3], including the CARDIGENE study in which no difference in HFE carrier frequency was found between DCM patients and controls [38]. If genetic predisposition is a cause of DCM in HC [39,40], there is limited understanding of the mechanism.

A third explanation for LV dysfunction in HC is the possibility of predisposition to autoimmune disease, as the HFE gene is located close to the human leucocyte antigen (HLA) locus. An autoimmune mechanism for DCM has been suggested in subjects where there is an abnormal response of the immune system to different insults [37,41]. The low CD8+ T cell count in DCM patients have been stated to support this hypothesis [41].

Therefore the four possible explanations for the LV dysfunction in HC, either alone or in concert, remain unresolved in relative importance. Our data from the current study substantially clarify this debate. We found 2 groups of *genetically confirmed* HFE-HC patients with LV dysfunction. The first group were those patients with myocardial siderosis (T2* <20 ms). The prevalence of myocardial siderosis at first presentation of HC in patients with serum ferritin >1000 ug/L was high at 33%. Of these *genetically confirmed* HFE-HC patients with myocardial siderosis, 83% had associated LV dysfunction and all had heart failure. Myocardial siderosis was therefore the commonest cause of LV dysfunction. A second group of *genetically confirmed* HFE-HC patients in our study with LV dysfunction had no myocardial iron loading but had well documented existing causes, namely anterior myocardial infarction, or substance abuse. Two other cases of LV dysfunction occurred in the *genetically unconfirmed* HC patients, which were caused by myocarditis or cancer treatment.

It was notable that evident coronary artery disease was not common in this predominantly middle-aged to elderly population, and there was little evidence of unexplained cardiomyopathy to support the other possible etiologies (direct genetic effects or autoimmunity) which have been previously postulated for LV dysfunction in HC. This indicates that myocardial T2* is required to definitively diagnose myocardial siderosis in newly presenting patients with HC. This has the added merit in patients with impaired or borderline LV function of confidently excluding myocardial iron loading as the cause where possible alternative diagnoses are known or suspected, such as coronary disease or the prior use of cardiotoxic drugs, as long-term specific treatment for the cause of LV dysfunction is required.

The limitations of this research include the relatively small sample size, which relates to the difficulty of recruiting newly-presenting HC patients. Late gadolinium enhancement was performed only in 7 patients in whom there was a possible history of cardiac disease and therefore there is limited data on cardiac fibrosis. Of the 5 *genetically confirmed* HFE-HC patients who presented in heart failure with a low cardiac T2*, 2 had LGE imaging with one having no LGE and one having mid-wall LGE with no prior history of cardiac pathology. It is not clear whether the latter patient’s LGE could be attributed to myocardial siderosis. Only 2 patients were referred to be rescanned for clinical reasons, and therefore the response of cardiac T2* to venesection is not known for all patients. However, in the 2 that were rescanned, there was an impressive improvement in myocardial T2* and EF with venesection over nearly 3 years in both cases. We did not test for nutritional factors including thiamine and vitamin D [42,43], which may exacerbate heart failure in other iron overload conditions such as thalassemia major, although there is little evidence for derangement in HC. The patient cohort comprised of patients with a clinical diagnosis of HC and subsequent genetic analysis was only able to confirm the genetic diagnosis in 31 of 41 patients studied. We present the data of these 31 *genetically confirmed* HFE-HC patients separately to ensure comparability with future studies. Further detailed genetic analysis of the 10 *genetically unconfirmed* HC patients was not available.

## Conclusion

In conclusion, our data show myocardial siderosis is common in HFE-HC patients (33%) presenting with iron overload (serum ferritin >1000 μg/L), a value associated with increased risk of liver damage in HFE-HC, and is uncommon below this threshold. Myocardial siderosis was the commonest cause of LV dysfunction, but some cases of heart failure were attributable to other causes as a result of the age of the population. There is little evidence for excess coronary artery disease, or a direct genetic or autoimmune cause for cardiomyopathy. In two cases of patients with heart failure and myocardial siderosis, we demonstrated a marked improvement in left ventricular function in parallel with an improvement in T2* following venesection. Therefore T2* CMR is not only useful for demonstrating the myocardial siderosis, but also in differentiating it from other causes of LV dysfunction which have different treatments and in monitoring the response to therapy.

## Abbreviations

HC: Genetic hemochromatosis; DCM: Dilated cardiomyopathy; CMR: Cardiovascular magnetic resonance; LV: Left ventricular; ROI: Region of interest; SD: Standard deviation; EF: Ejection fraction; LGE: Late gadolinium enhancement; CAD: Coronary artery disease; EDV: End diastolic volume; ESV: End systolic volume; BSA: Body surface area; LDL: Low density lipoprotein.

## Competing interests

Dr Grasso and Dr Dooley have no relevant conflicts of interest to disclose.

## Authors’ contributions

JPC and AEG collected and analysed the data and drafted the manuscript. JBP, FS and JD collected the data and critically reviewed the manuscript. DJP conceived and designed the study, analysed the data and drafted the manuscript. All authors read and approved the final manuscript.

## Financial support

This study was supported by the UK National Institute of Health Research Cardiovascular Biomedical Research Unit of Royal Brompton & Harefield NHS Foundation Trust and Imperial College London.

## Relationships with industry

Professor Pennell is a consultant to Novartis, Siemens and Apo Pharma, and is a director and stockholder in Cardiovascular Imaging Solutions. Dr Carpenter has received speakers’ honoraria from Swedish Orphan and Apo Pharma. Professor Porter is a consultant to and receives research funding from Novartis. Dr Shah has received speaker’s honoraria from Novartis and Apo Pharma.
